# Autophagy crosstalk with the immune microenvironment in chronic myeloid leukemia and serves as a biomarker for diagnosis and progression

**DOI:** 10.3389/fimmu.2025.1570903

**Published:** 2025-05-28

**Authors:** Fangmin Zhong, Fangyi Yao, Jing Liu, Qun Fang, Xiajing Yu, Bo Huang, Xiaozhong Wang

**Affiliations:** Jiangxi Province Key Laboratory of Immunology and Inflammation, Jiangxi Provincial Clinical Research Center for Laboratory Medicine, Department of Clinical Laboratory, The Second Affiliated Hospital, Jiangxi Medical College, Nanchang University, Nanchang, Jiangxi, China

**Keywords:** chronic myeloid leukemia, autophagy, immune microenvironment, molecular subtypes, machine learning, diagnosis

## Abstract

**Background:**

Previous studies have shown that autophagy is closely related to the occurrence, development, and treatment resistance of chronic myeloid leukemia (CML) and has dual roles in promoting cell survival and inducing cell death.

**Methods:**

We analyzed autophagy levels in CML samples via transcriptome data and evaluated the relationships between autophagy and the immune microenvironment, treatment response, and disease progression. A consensus clustering algorithm was used to identify autophagy-related molecular subtypes. The value of autophagy-related genes (ARGs) in diagnosis and treatment evaluation was analyzed and verified by a variety of machine learning algorithms.

**Results:**

Compared with normal samples, CML samples had significantly lower autophagy scores and more downregulated ARGs. The autophagy score was positively correlated with the activity of immune and signal transduction-related pathways and negatively correlated with proliferation-related pathways. Patients with high autophagy scores had a greater proportion of regulatory T-cell infiltration and greater cytokine–cytokine receptor interaction signaling pathway activity, while patients with low autophagy scores had greater γδT cell infiltration and PD-1 expression. Low autophagy scores are also associated with malignant progression and nonresponse to treatment. The immune landscape and chemotherapy sensitivity significantly differed between the two autophagy-related molecular subtypes. Three diagnostic ARGs (FOXO1, TUSC1, and ATG4A) were identified by support vector machine recursive feature elimination, least absolute shrinkage selection operator, and random forest algorithms, and the combined diagnostic efficiency of the three was further improved. The diagnostic value of the three ARGs was verified by an additional validation cohort and our clinical real-world clinical cohort, and they can also be used for the differential diagnosis of CML from other hematological malignancies.

**Conclusion:**

Our study revealed that CML samples exhibit decreased autophagy, and autophagy may induce Tregs to undergo immunosuppression through cytokines. Autophagy-related molecular subtypes are helpful for guiding the clinical treatment of CML. The identification of ARGs by a variety of machine learning algorithms has potential clinical application value.

## Introduction

Chronic myeloid leukemia (CML) is a myeloproliferative disease arising from the malignant transformation of hematopoietic stem cells (1). The Philadelphia chromosome is formed by reciprocal translocation of chromosomes 9 and 22, resulting in a new fusion gene, BCR-ABL, which encodes a protein with strong tyrosine kinase activity, leading to the malignant proliferation of CML cells (2). Tyrosine kinase inhibitors (TKIs), such as imatinib, dasatinib, and nilotinib, have shown remarkable efficacy, and the survival time of CML patients has significantly increased (3). However, due to the existence of various primary and secondary resistance factors, some patients have poor responses to TKI treatment (4). Therefore, exploring new biological targets is helpful for promoting the development of personalized treatments for CML.

Autophagy is an intracellular catabolic process in which damaged organelles and cytoplasmic contents, such as protein aggregates, are encapsulated in autophagosomes for lysosomal degradation to meet the metabolic needs of cells and the renewal of organelles (5, 6). Autophagy is widely involved in physiological and pathological processes in the body and has been implicated in the pathogenesis of a variety of human diseases, including cancer (7). However, autophagy has a dual role in tumor cells (8, 9). On the one hand, it can induce autophagic cell death (10), and on the other hand, it can maintain cell homeostasis and protect cells from harmful factors, which is conducive to cell survival (11, 12). At present, the use of autophagy as a target for intervention has been controversial. Similar to that in most tumors, autophagy plays two roles in CML. The antitumor effects of pristimerin were observed to be mediated through the induction of autophagy via excessive generation of ROS and activation of the JNK signaling pathway. This mechanism leads to cell cycle arrest, inhibition of cell proliferation, and induction of autophagic cell death in the CML cell line K562 (13). Another study showed that the NF-κB inhibitor bardoxolone methyl promoted autophagy and induced apoptosis in K562 cells by regulating the PI3K/AKT/mTOR and p38 MAPK/ERK1/2 signaling pathways (14). In addition, curcumin can induce apoptosis in K562 cells by inducing autophagy (15). Imatinib can also inhibit the PI3K/AKT/FOXO4/ATF5/mTOR pathway to induce autophagy by inhibiting the BCR-ABL protein (16). In terms of cancer promotion, knockdown of the Beclin1 gene in a mouse CML model reduced leukemia burden by inhibiting autophagy (17). Autophagy promotes leukemogenesis and cell survival by inhibiting cellular stress (18). Autophagy is closely related to the occurrence and development of CML. However, its exact role in the immune microenvironment of CML and whether autophagy-related genes (ARGs) can be used as biomarkers for the diagnosis and prognosis evaluation of CML remain unknown.

With the advancement of biotechnology, the utilization of machine learning algorithms can effectively contribute to disease diagnosis and treatment decision-making (19). Consequently, machine learning holds significant clinical value across various healthcare systems. Currently, the implementation of machine learning in CML remains limited. Although several studies have developed machine learning models for CML diagnosis using peripheral blood smear or bone marrow puncture images, the absence of external validation diminishes model reliability (20, 21). Therefore, employing machine learning algorithms to identify additional diagnostic markers for CML through multicohort studies would greatly benefit early detection and personalized treatment guidance.

In this study, we explored the association between the level of autophagy and the immune microenvironment of CML patients by bioinformatics analysis. The identification of molecular subtypes based on ARG expression is helpful for the establishment of personalized treatment regimens for CML. In addition, by using multiple machine learning algorithms, we identified a set of ARGs that accurately diagnose CML, which were validated in additional public and clinical cohorts. These results provide new insights for the study of autophagy in CML.

## Methods

### Data collection and preprocessing

The CML cohorts GSE13159 and GSE144119 were downloaded from the GEO database. The GSE13159 cohort consisted of 76 CML samples and 74 normal samples, which were standardized by downloading the “cel” file and used as the analysis cohort for this project. The GSE144119 cohort consisted of 48 samples from newly diagnosed CML patients and 32 samples from CML patients in remission, as well as 17 normal samples, and the data were converted to transcripts per kilobase million (TPM) values for subsequent validation. In addition, we collected 10 CML samples and 5 normal samples for transcriptome sequencing. Sample collection was approved by the Second Affiliated Hospital of Nanchang University, and patient consent was obtained. Ethical approval was obtained in accordance with the guidelines of the Declaration of Helsinki. The data from our clinical cohort were similarly transformed into TPM values for subsequent validation. Standardized RNA-seq data (TPM values) of 173 TCGA-LAML (The Cancer Genome Atlas-Acute Myeloid Leukemia) samples were downloaded from the UCSC Xena database (https://xenabrowser.net/datapages/). In addition, GSE13159 contained 750 acute lymphoblastic leukemia samples, 542 acute myeloid leukemia samples, 448 chronic lymphocytic leukemia samples, and 206 myelodysplastic syndrome samples, which were further used in the differential diagnosis of CML. The GSE44589 and GSE14671 cohorts contained sequencing data of 198 and 59 imatinib-treated samples, respectively, for the evaluation of CML treatment response. A total of 232 ARGs were extracted from the human autophagy database (http://www.autophagy.lu/index.html).

### Differential expression analysis of ARGs

ARGs with differential expression between CML and normal samples were screened by the “limma” package. Genes with a |logFC>0.5| and adjusted P value<0.05 were considered differentially expressed ARGs (DEARGs). Subsequently, we performed Gene Ontology (GO) annotation and Kyoto Encyclopedia of Genes and Genomes (KEGG) pathway enrichment analysis of these genes using the “clusterProfiler” package (22). We quantified the activity of a pathway or biological pathway by calculating an enrichment score for a gene set using the gene set variation analysis (GSVA) algorithm. The GSVA algorithm initially ranks the expression levels of all genes within a single sample in descending order, followed by an analysis of the positioning of target gene sets within this ranking. If these genes exhibit high expression levels, they will be ranked higher, indicating elevated activity of the corresponding gene set or pathway. In this study, we assessed the scores of the autophagy gene set as a representation of autophagic activity within each sample.

### Correlation analysis and protein–protein interaction network construction

Spearman correlation analysis was used to analyze the correlations between DEARGs. The STRING database (https://string-db.org/) was used to analyze the protein–protein interactions of the DEARGs. The cutoff criteria were set as follows: a minimum confidence level of 0.7 was needed, while all other settings remained at their default values. Then, Cytoscape software was used to visualize the PPI network.

### Weighted correlation network analysis

The “WGCNA” software package was used to identify genes related to autophagy scores in the GSE13159 cohort (23). Pearson correlation analysis was used to construct the adjacency matrix of all matched genes, and the scale-free topology of the adjacency matrix was implemented based on the optimal soft threshold power. Then, the adjacency matrix is transformed into a topological overlap matrix (TOM) to reduce noise and false correlation, thereby obtaining a refined distance matrix (24). Based on the TOM dissimilarity measure, the minimum module size was set to 30, the cut height was set to 0.2, and genes with similar expression patterns were divided into the same modules by average linkage hierarchical clustering. Then, the correlation between module eigengenes (MEs) and the autophagy score was evaluated.

### Estimation of immune cell infiltration

To characterize immune cell infiltration in CML samples, we used a deconvolution algorithm, CIBERSORT, to quantify the proportions of 22 immune cell types based on individual sample gene expression profiles (25).

### Identification of molecular subtypes based on ARG expression

To better evaluate the individual differences in CML patients, we used the “consensusclusterplus” package to perform cluster analysis of CML samples based on the expression profiles of DEARGs to identify CML subtypes. The results of the cluster analysis were reliable and stable after 1000 iterations. The PCA algorithm was used to verify the classification. The first two principal components were selected based on the magnitude of the eigenvalues to effectively capture the majority of the data variation. A scatter plot was then generated to visually depict the projection of samples onto these principal components, facilitating an intuitive understanding of sample positioning and cluster formation.

### Prediction of the sensitivity of CML samples to TKI treatment

The expression matrix and drug response data of blood cell lines from the Cancer Genome Project (CGP) database (https://cancer.sanger.ac.uk/cosmic) were used to predict the half-maximal inhibitory concentrations (IC50) of TKIs in CML samples via the “pRRophetic” package (26).

### Identification of diagnostic biomarkers for CML

Three machine learning algorithms, support vector machine recursive feature elimination (SVM-RFE), least absolute shrinkage selection operator (LASSO), and random forest (RF) (19), were used to screen the diagnostic ARGs in CML. We used the “glmnet” package, the “e1071” package, and the “randomForest” package for LASSO, SVM, and RF analyses, respectively, with fivefold cross-validation in the analysis cohort. In addition, regression coefficients for diagnostic ARGs were calculated by LASSO regression analysis, and a CML risk score diagnostic model was constructed based on the following formula:


$$Risk\;score=\;\sum_{1}^{i}\left(Coefi*ExpGenei\right),$$

where i is the diagnostic ARG and “Coef” and “ExpGene” are the regression coefficient and the expression value of the ARG, respectively (**Supplementary Table S1**). Through the construction of the risk score model, we can further analyze the combined diagnostic value of ARGs.

### Construction of the miRNA regulatory network for CML diagnostic ARGs

We used the miRTarBase, miRDB, and TargetScan databases to predict miRNAs with binding sites for CML diagnostic ARGs and screened out miRNA–target pairs predicted by all three databases. The GSE90773 cohort contained data on differentially expressed miRNAs between CML cells and normal cells and was used for the construction of a miRNA regulatory network.

### Statistical analysis

All analyses were performed with R software and corresponding software packages. Differences between two or more groups were analyzed using the Wilcoxon rank sum test and the Kruskal–Wallis test, respectively. The diagnostic value of biomarkers was determined by receiver operating characteristic (ROC) curve analysis. A bilateral adjusted P<0.05 (Bonferroni correction) indicated a significant difference.

## Results

### Autophagy-related molecular characteristics in CML

**Figure 1:**
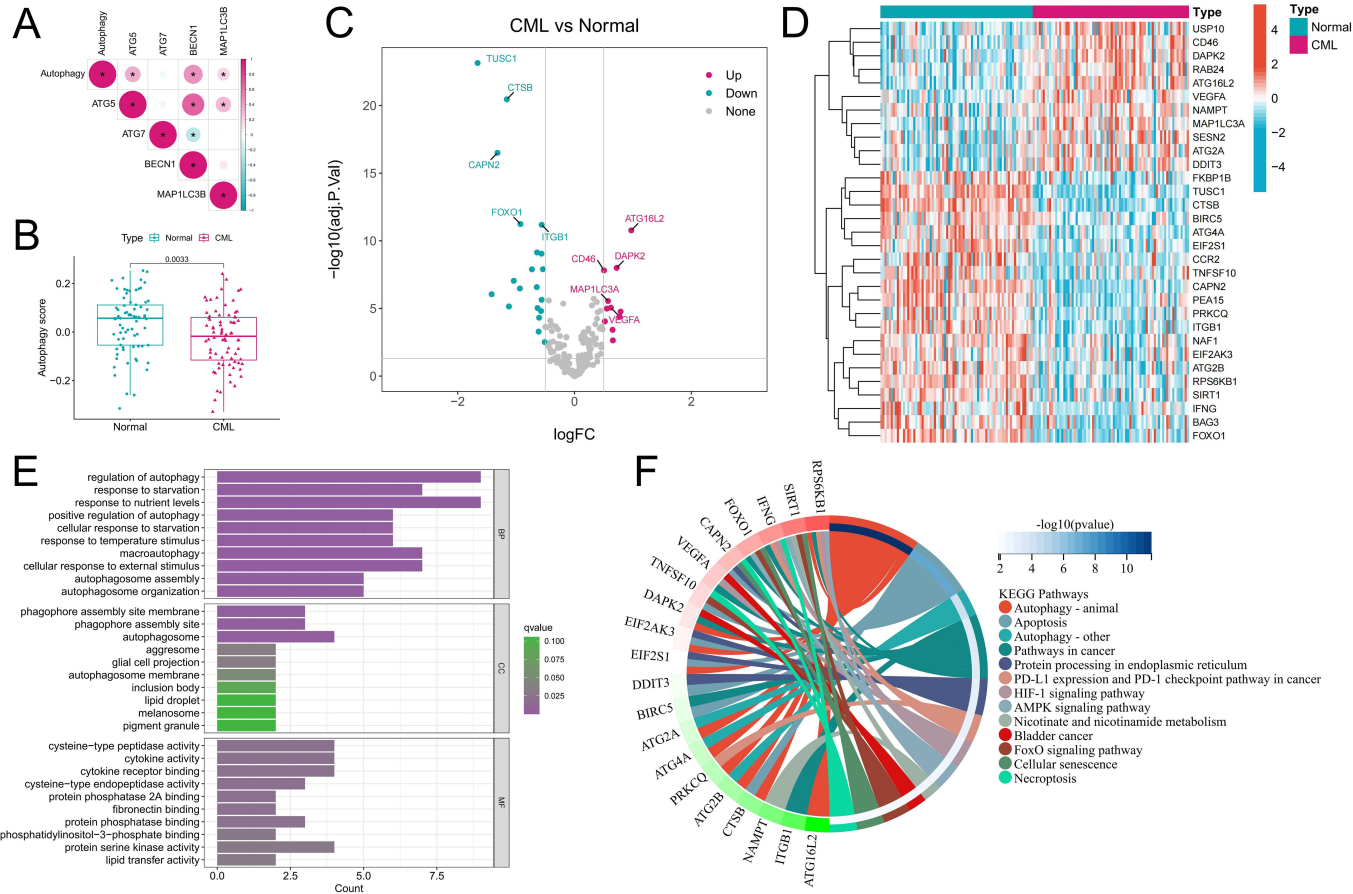
Characteristics of autophagy activity and ARG expression in CML samples. **(A)** Correlation analysis of the autophagy score and autophagy marker gene expression. Red indicates a positive correlation, blue indicates a negative correlation, and the darker the color is, the stronger the correlation. *P<0.05. **(B)** Autophagy score distribution in normal (n=74) and CML (n=76) samples. **(C, D)** Volcano map **(C)** and heatmap **(D)** showing the expression characteristics of ARGs. **(E, F)** Functional annotation **(E)** and pathway enrichment analysis **(F)** of DEARGs. The functional enrichment analysis in Figure F highlights the most significant signaling pathways and their corresponding genes, while excluding the visualization of less enriched pathways and their associated genes. BP, biological process; CC, cellular component; MF, molecular function. (*P < 0.05).

The activity and molecular characteristics of autophagy in CML were systematically evaluated by calculating an autophagy score using the GSVA algorithm. Correlation analysis revealed that the autophagy score was significantly positively correlated with the expression of classic autophagy marker genes, such as ATG5, BECN1, and MAP1LC3B (P<0.05) (**Figure 1A**), indicating that the autophagy score could reflect autophagy activity to a certain extent. The significantly lower autophagy score in CML samples than in normal samples may indicate that CML cells are resistant to autophagic death (**Figure 1B**). **Figures 1C, D** show the expression characteristics of 31 DEARGs in CML samples and normal samples, among which CML samples had more downregulated ARGs. The biological functions of the DEARGs included mainly the regulation of autophagy, phagophore assembly site membrane, cysteine-type peptidase activity and cytokine activity (**Figure 1E**). In addition to autophagy, they are involved in biological pathways such as apoptosis, pathways in cancer, protein processing in the endoplasmic reticulum, PD-L1 expression and the PD-1 checkpoint pathway in cancer, and the HIF-1 signaling pathway (**Figure 1F**). These results suggest that autophagy in CML samples is perturbed and may have an impact on the occurrence and development of CML.

### Correlations between the expression of DEARGs and the autophagy score and immune characteristics

**Figure 2:**
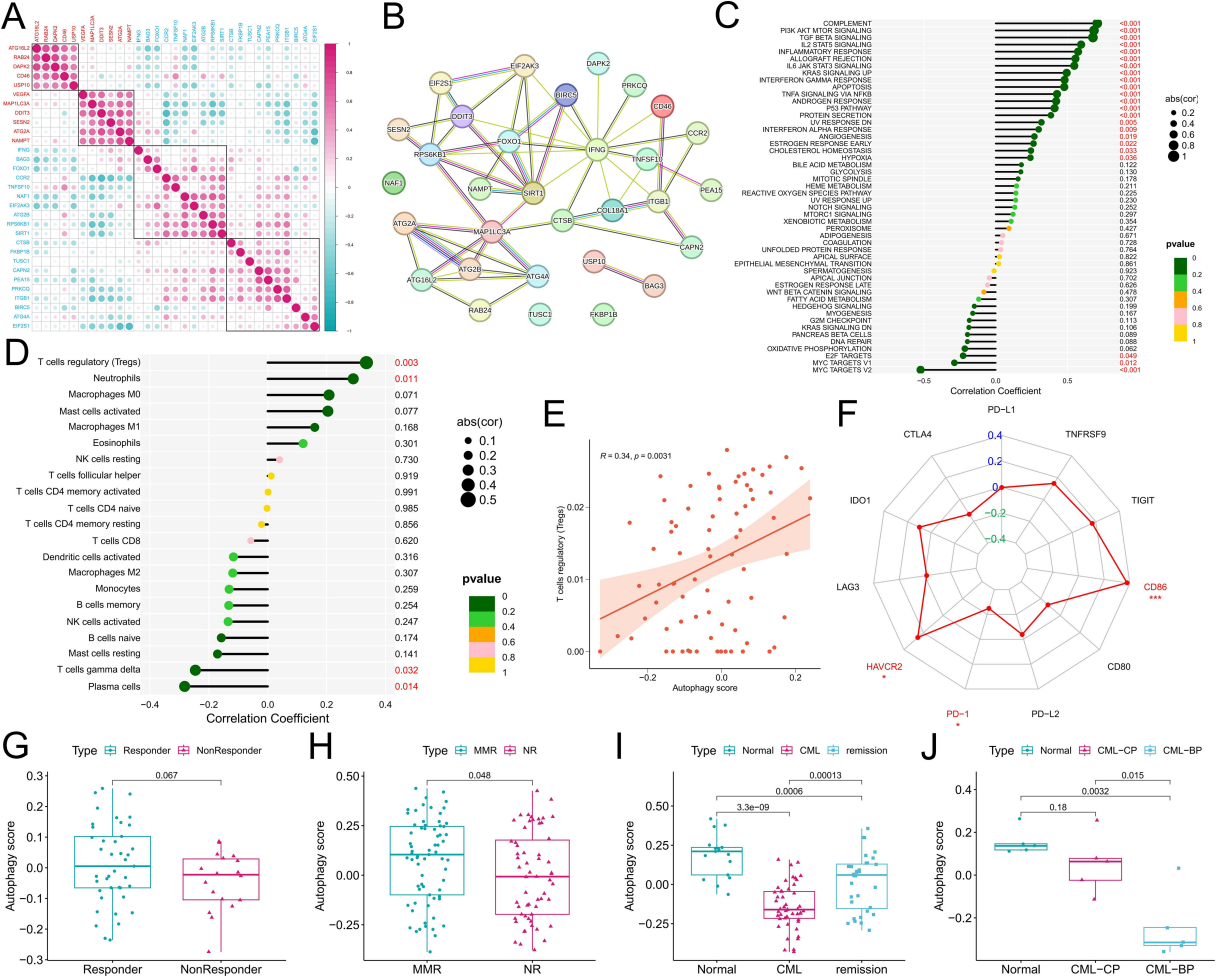
Correlations of the autophagy score with the immune microenvironment, treatment response, and disease progression in CML patients. **(A)** Expression correlation analysis among DEARGs; red indicates the upregulated expression of DEARGs in CML, and blue indicates the downregulated expression. **(B)** PPI network analysis of the DEARGs. The solid lines usually represent direct physical interactions, such as the binding between proteins. Different colors represent different interaction types or functional categories. The specific color meanings can be referred to the STRING database. **(C-F)** Correlation analysis of the autophagy score with the tumor marker gene set enrichment score **(C)**, immune cell infiltration **(D)**, Treg infiltration **(E)**, and immune checkpoint expression **(F)**. **(G, H)** Differences in autophagy scores between patients in the GSE14671 **(G)** and GSE44589 **(H)** cohorts who responded to TKI treatment and those who did not. **(I)** Differences in autophagy scores among normal, CML, and treatment-remission samples in the GSE144119 cohort. **(J)** Differences in autophagy scores among normal, chronic-phase (CP), and blast-phase (BP) CML samples in our clinical cohort. MMR, major molecular response; NR, no response. P values refer to adjusted P values. (*P < 0.05; ***P < 0.001).

Expression correlation analysis among the DEARGs revealed a positive correlation among the upregulated ARGs in CML, and there was also a positive correlation among the downregulated ARGs (**Figure 2A**), indicating that ARGs play a synergistic role in CML to jointly regulate autophagy in CML cells. The PPI network showed that IFNG, SIRT1, FOXO1, and MAP1LC3A were the core genes in these DEARGs (**Figure 2B**). Among them, MAP1LC3A, whose expression is upregulated, may be a key positive regulator of autophagy in CML cells, while IFNG, SIRT1, and FOXO1 may play important inhibitory roles. We further analyzed the relationships between the autophagy score and cancer marker signaling pathway activity and the immune microenvironment to reveal the underlying biological mechanism of autophagy in CML. We found that autophagy scores were significantly and positively correlated with enrichment scores for multiple immune pathways, such as complement, inflammatory response, allograft rejection, and IL6/JAK/STAT3 signaling, and multiple signaling pathways, such as PI3K/AKT/mTOR signaling, TGF beta signaling, and IL2/STAT5 signaling (**Figure 2C**). In addition, the autophagy score was also significantly positively correlated with the apoptosis score and negatively correlated with proliferation-related pathways such as the E2F target and MYC target V1/V2 scores, indicating that CML cell-mediated autophagy may induce apoptosis and inhibit proliferation, again indicating that a lower autophagy score in CML cells may be related to apoptosis resistance and malignant proliferation. Immune infiltration analysis revealed that the autophagy score was significantly positively correlated with the infiltration of regulatory T cells (Tregs) and neutrophils and significantly negatively correlated with γδT cells and plasma cells (**Figures 2D, E**), indicating that increased autophagy in CML cells may be related to immunosuppression and that Tregs may play an important role in this process. We also noted that higher autophagy scores were accompanied by higher HAVCR2 and CD86 expression and lower PD-1 expression (**Figure 2F**).

### Correlation of the autophagy score with therapeutic response and disease progression

Analysis of treatment data from the GSE14671 and GSE44589 cohorts revealed that autophagy scores were significantly greater in patients who responded to imatinib treatment than in those who did not (P<0.05) (**Figures 2G, H**). It is worth noting that for **Figure 2G**, despite the p value exceeding 0.05, there still exist discernible disparities in the data distribution between the two groups. In addition, in the GSE144119 cohort, we observed a significant increase in autophagy scores in CML patients who responded to TKIs such as imatinib (**Figure 2I**). In our clinical cohort, we observed that the autophagy score gradually decreased with the progression of CML from the normal group to the chronic-phase CML group and then to the blast-phase CML group (**Figure 2J**), where CML in the blast phase indicated no response to TKI treatment. As shown in **Figure 2J**, although our limited clinical sample size allowed for an outlier resulting in a P value > 0.05, the overall trend remained unaltered, and these findings may also reflect corresponding biological patterns and provide supplementary evidence. Therefore, multiple sets of data have confirmed that a low autophagy score is related to CML occurrence, malignant progression, and nonresponse to TKI treatment.

### Coexpression gene networks potentially associated with CML autophagy were identified by WGCNA

**Figure 3:**
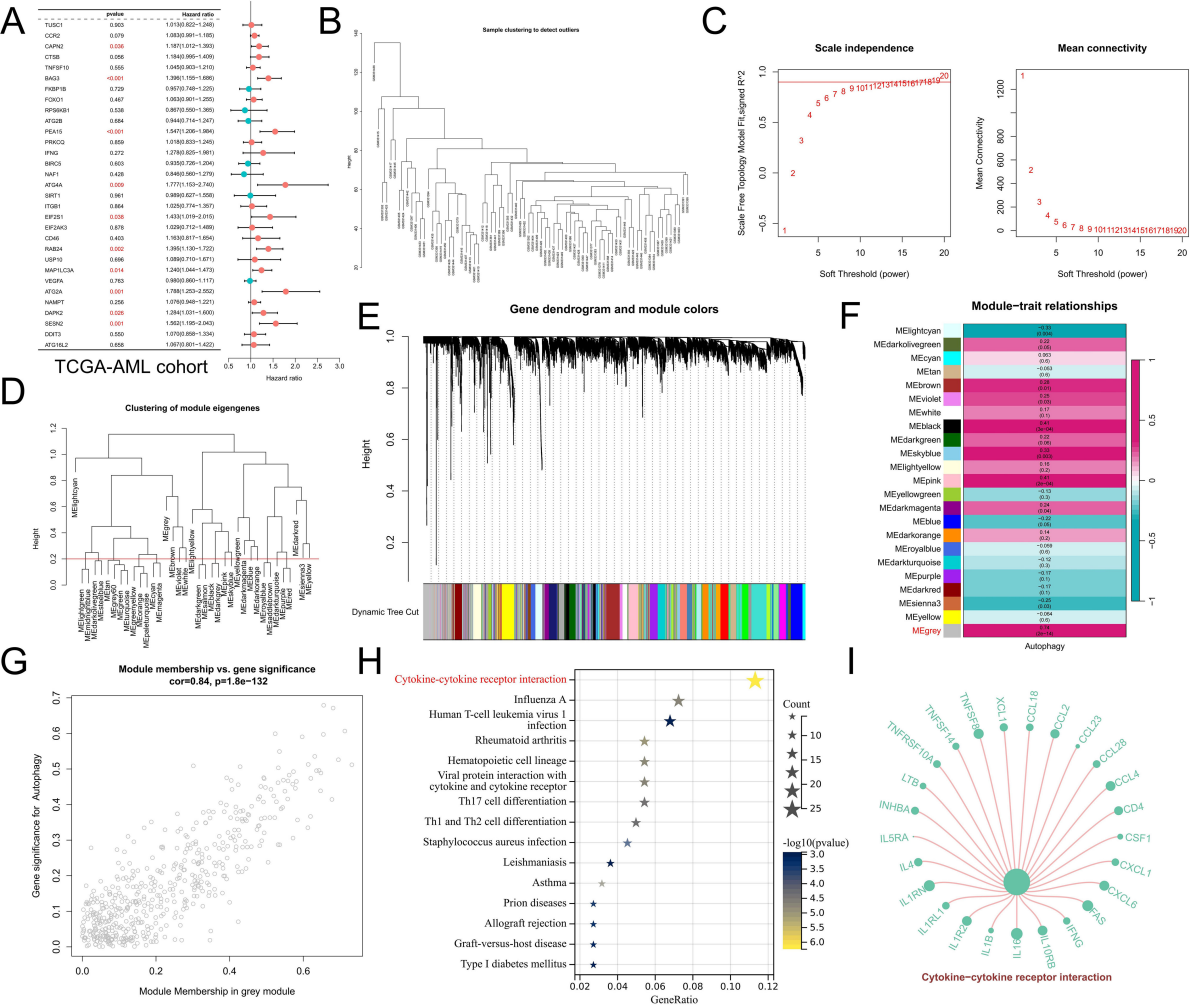
WGCNA revealed potential mechanisms of autophagy regulation. **(A)** Univariate Cox regression analysis revealed the prognostic features of the DEARGs in the TCGA-AML cohort. **(B)** Cluster plot of CML samples. **(C)** Analysis of various soft threshold powers using the scale-free fitting index and average connectivity. The abscissa of both figures represents the value of the soft threshold (power). The ordinate of the left figure is the scale-free fit index, that is, the signed R^2^. The greater the square of the correlation coefficient is, the closer the network is to the distribution of the scale-free network. When the signed R^2^ is greater than 0.9, the network conforms to the distribution of the scale-free network. There is a red horizontal line in the figure, which indicates the best power value when the first signed R2 reaches this red line, which is 12 in this figure. The ordinate of the right graph represents the average connectivity number of all nodes, and a lower ordinate indicates better connectivity. **(D)** Clustering of different modules. The red line is the cutting height (0.2) to merge the modules with a correlation greater than 0.8. **(E)** Cluster plots based on different measures (1-TOM). **(F)** Heatmap of correlations between module genes and autophagy scores. **(G)** Scatter plot of module genes associated with the autophagy score in gray modules. **(H)** KEGG enrichment analysis of gray module genes. **(I)** Genes enriched in the cytokine–cytokine receptor interaction signaling pathway in the gray module. The size of the point represents the correlation of the specified gene with the corresponding phenotype, and the larger the point is, the greater the correlation.

In addition, considering that approximately 70% of CML patients in the blast phase progress to AML, we analyzed the prognostic predictive value of the DEARGs in the TCGA-AML cohort. Univariate Cox regression analysis revealed that ARGs significantly related to the prognosis of AML patients were prognostic risk factors (P<0.05, hazard ratio>1) (**Figure 3A**), indicating that they may be involved in the malignant progression of AML. Moreover, to better explore potential mechanisms associated with autophagy in CML, we investigated the coexpressed gene networks significantly associated with autophagy scores using WGCNA in the GSE13159 analysis cohort. The cluster tree diagram shows the clustering characteristics of the CML samples (**Figure 3B**). **Figures 3C, D** show the scale-free fit exponentials and average connectivity analyses for various soft threshold powers. We set the cutoff height = 0.2 to merge the modules with a correlation greater than 0.8 (**Figure 3E**). According to the optimal soft threshold power β=12 (unscaled R^2^ = 0.9), the 5000 genes with the highest standard deviation were divided into 23 independent coexpression modules (**Figure 3F**). The correlogram of the module-trait relationship showed that the gray module had the highest correlation with the autophagy score (Cor=0.74, P=2e-14). In addition, the greater the correlation between the genes and the gray module was, the greater the correlation with the autophagy score, confirming that the gray module genes are strongly correlated with CML autophagy (**Figure 3G**). Through KEGG enrichment analysis, we found that gray module genes were significantly enriched in the cytokine–cytokine receptor interaction signaling pathway (**Figures 3H, I**). Given the significant positive correlation between Treg infiltration and autophagy scores, we hypothesized that CML autophagy-induced cytokine secretion may exert immunosuppressive effects by promoting Treg infiltration.

### Identification of autophagy-related molecular subtypes and analysis of differences in biological characteristics between subtypes

**Figure 4:**
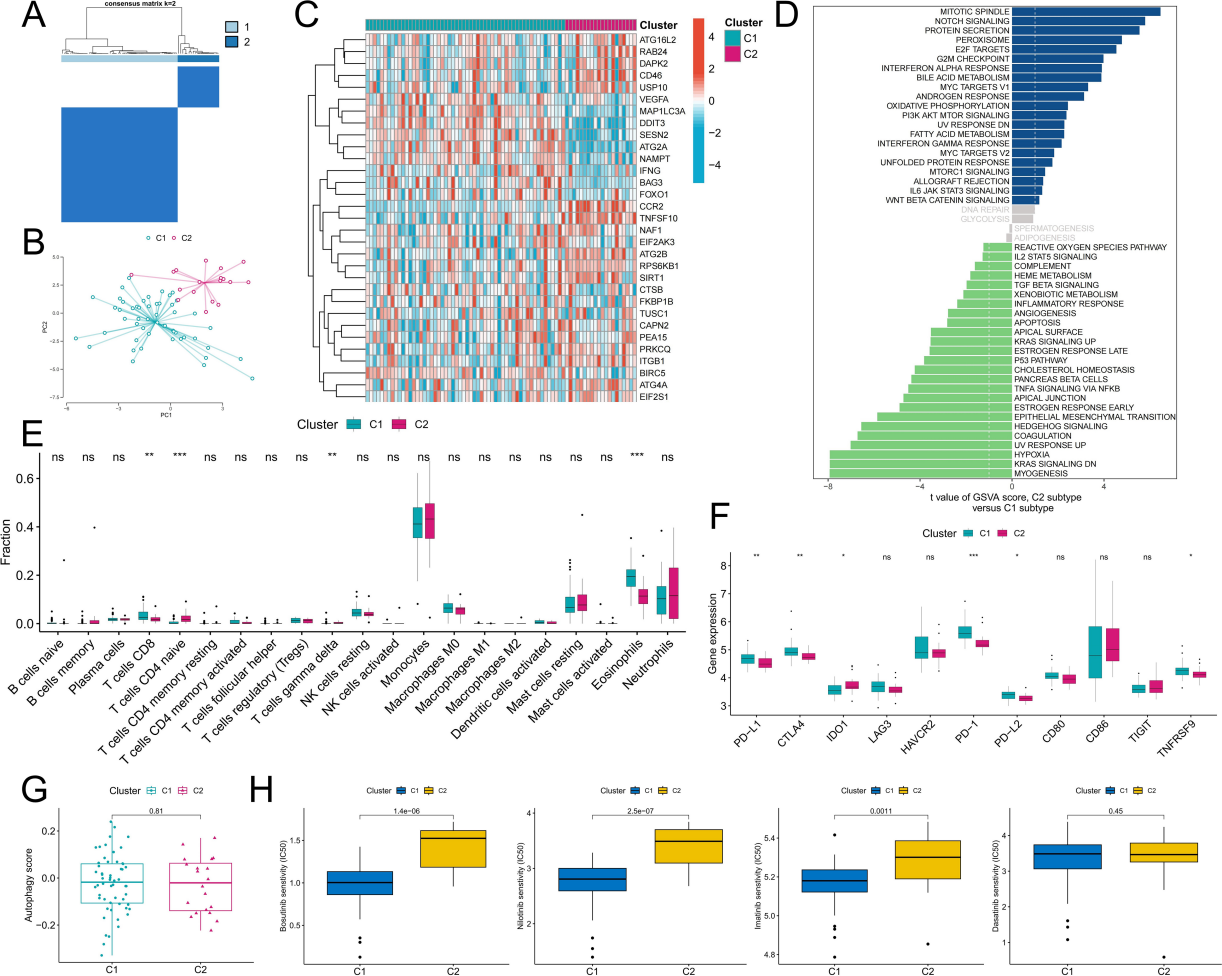
Identification of autophagy-related molecular subtypes and analysis of their differences in biological characteristics and chemotherapy sensitivity. **(A)** Based on the expression of DEARGs, CML patients were divided into two autophagy-related molecular subtypes by a consensus clustering algorithm. **(B)** The PCA algorithm was used to analyze the differences in the distribution of patients between subtypes. **(C-F)** Differences in the expression of DEARGs **(C)**, activity of tumor hallmark gene sets **(D)**, infiltration of 22 immune cells **(E)**, expression of immune checkpoints **(F)**, autophagy scores **(G)**, and therapeutic sensitivity to four TKIs **(H)** between the two MSs. (ns: P > 0.05; *P < 0.05; **P < 0.01; ***P < 0.001).

To better analyze the biological value of ARGs in CML, two CML molecular subtypes, Cluster C1 and Cluster C2, were identified by a consensus clustering algorithm in CML samples of the GSE13159 cohort based on the expression of DEARGs (**Figure 4A**). PCA confirmed that the two molecular subtypes had significantly different distribution characteristics (**Figure 4B**). The heatmap showed that ARGs such as VEGFA, MAP1LC3A, DDIT3, and SESN2 were upregulated in the C1 subtype (**Figure 4C**) and were similarly upregulated in CML samples compared to normal samples. To further evaluate the differences in biological characteristics between the two subtypes (**Figure 4D**), we first performed GSVA and found that the C2 subtype had increased activity of proliferation-related pathways such as the mitotic spindle, E2F targets, G2M checkpoint, and MYC target V1. Subtype C1 was enriched in myogenesis, KRAS signaling DN, hypoxia, and other signaling pathways. In addition, immune infiltration analysis revealed that the C1 subtype contained a greater proportion of CD8+ T cells and eosinophils, while the C2 subtype contained more naive CD4+ T cells and γδT cells (**Figure 4E**). The expression of immune checkpoint molecules also significantly differed between the two subtypes. The expression of immune checkpoint molecules was also significantly different between the two subtypes, among which the expression of PD-L1, CTLA4, PD-1, PD-L2, and TNFRSF9 was significantly upregulated in the C1 subtype (**Figure 4F**). These features suggest that the C1 subtype may be subject to some immunosuppression, resulting in the inability of CD8+ T cells to effectively exert their killing effects. Subsequently, we observed no significant difference in autophagy scores between the two subtypes (**Figure 4G**). Drug prediction analysis revealed that the C1 subtype was more sensitive to imatinib, nilotinib, and bosutinib, and there was no significant difference in the IC50 of dasatinib between the two subtypes (**Figure 4H**). These results can guide personalized treatment of CML.

### Analysis of the diagnostic value of ARGs

**Figure 5:**
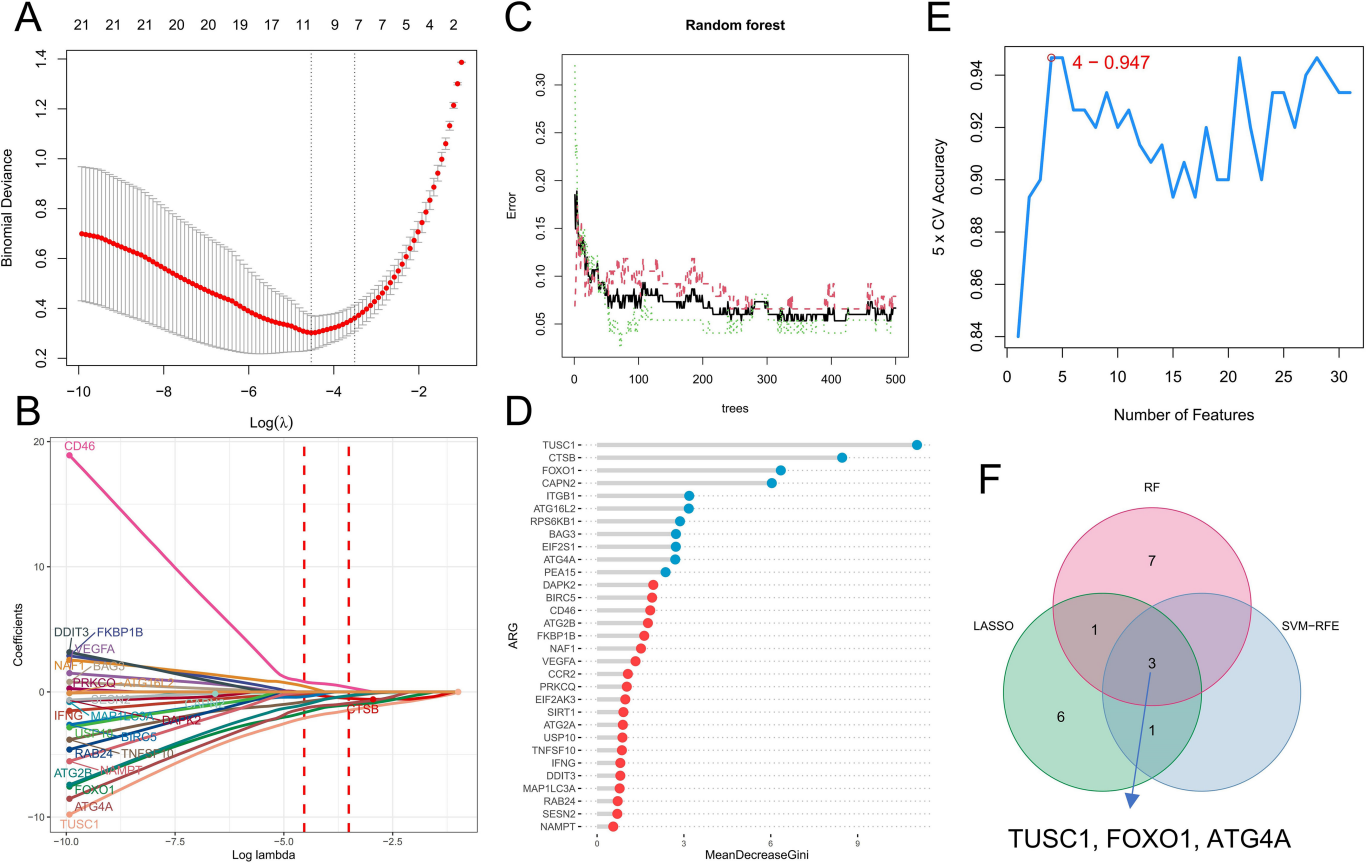
Identification of diagnostic ARGs. **(A, B)** Diagnostic ARGs were identified by the LASSO regression algorithm. The logarithm of the best tuning parameter (log lambda) was selected by cross-validation in the LASSO regression analysis, corresponding to the point with the smallest binomial deviance **(A)**. The model genes with nonzero coefficients and their corresponding coefficients were screened based on the best log lambda value **(B)**. **(C, D)** Diagnostic ARGs were identified by the RF algorithm. The red line represents the error in the CML group, the green line represents the error in the normal group, and the black line represents the total sample error. Analysis was performed based on minimum error points corresponding to 410 optimal random forest trees **(C)**. MeanDecreaseGini shows the rank of genes according to their relative importance, and genes with MeanDecreaseGini scores greater than 2 were further screened **(D)**. **(E)** The SVM-RFE algorithm was used to calculate the accuracy of fivefold cross-validation for different gene combinations, where the highest accuracy was achieved when the number of genes was 4. **(F)** Venn diagram of variables identified by the LASSO, RF, and SVM-RFE algorithms.

**Figure 6:**
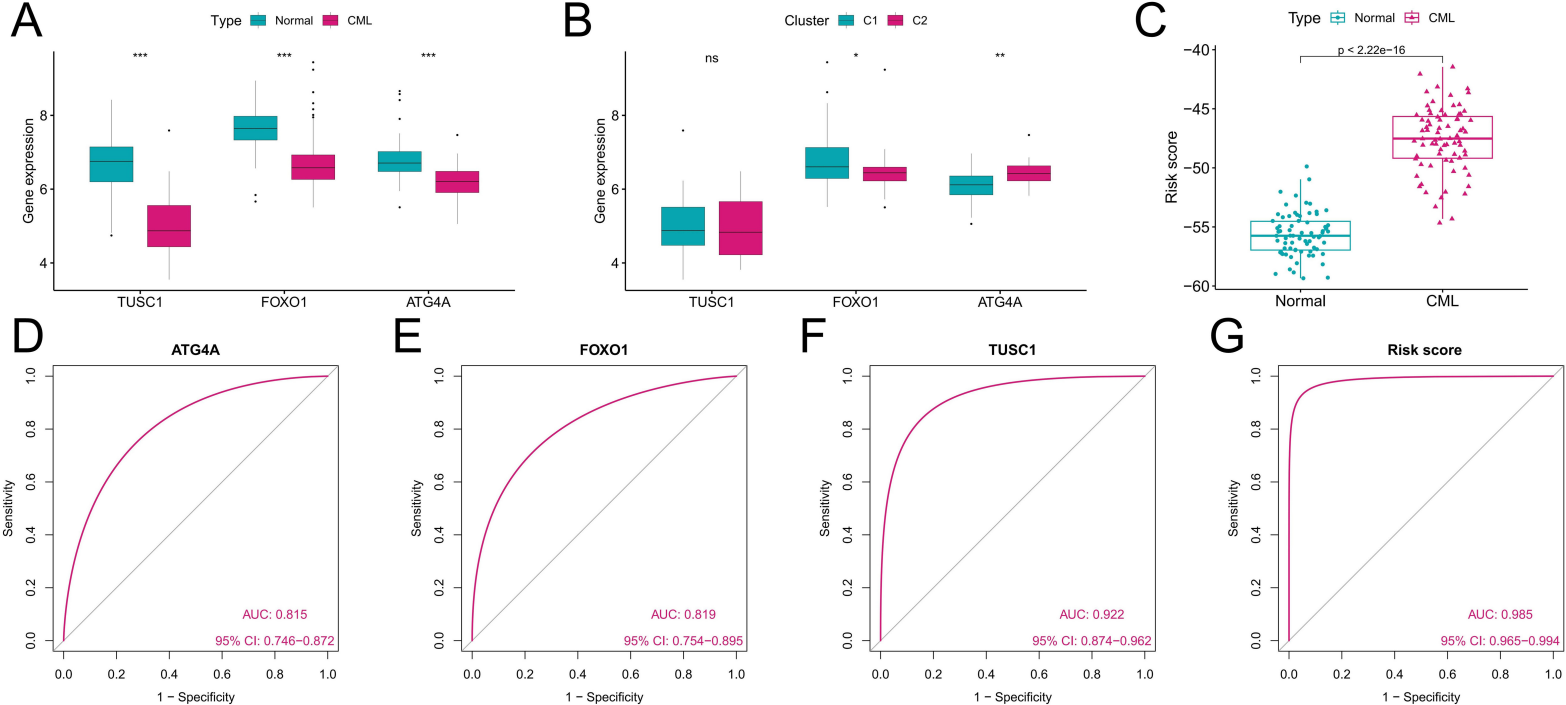
Analysis of the diagnostic value of the diagnostic ARGs. **(A)** Differences in the expression of the three diagnostic ARGs between CML samples and normal samples in the GSE13159 cohort. **(B)** Differences in the expression of the three diagnostic ARGs between autophagy-related molecular subtypes. **(C)** Differences in the risk score between CML samples and normal samples in the GSE13159 cohort. **(D-G)** ROC curve analysis was used to evaluate the diagnostic value of the three ARGs and the risk score in the GSE13159 cohort. (ns: P > 0.05; *P < 0.05; **P < 0.01; ***P < 0.001).

We further analyzed the diagnostic value of ARGs in CML. Three machine learning algorithms (LASSO, RF, and SVM-RFE) were used for dimensionality reduction, and we identified 11, 11, and 4 variables associated with CML from DEARGs, respectively (**Figures 5A–E**), including three overlapping diagnostic ARGs (TUSC1, FOXO1, and ATG4A) (**Figure 5F**). All three ARGs were significantly downregulated in CML samples compared with normal samples (P<0.05) (**Figure 6A**). There was no significant difference in TUSC1 expression between the two molecular subtypes, while FOXO1 was expressed at a greater level in the C1 subtype, and ATG4A was expressed at a greater level in the C2 subtype (P<0.05) (**Figure 6B**). We constructed a risk score model based on the three diagnostic ARGs by LASSO regression analysis to analyze their combined diagnostic value. CML samples had significantly greater risk scores than normal samples (**Figure 6C**). ROC curve analysis revealed that the AUCs of TUSC1, FOXO1, and ATG4A and the risk score model were 0.815, 0.819, 0.922, and 0.985, respectively (**Figures 6D–G**). The specificity of the risk score was 0.949 and its sensitivity was 0.932, indicating that these three ARGs had high diagnostic value and that their combined diagnostic value was further improved.

### Validation of the diagnostic value of ARGs and analysis of their role in the evaluation of therapeutic effects

**Figure 7:**
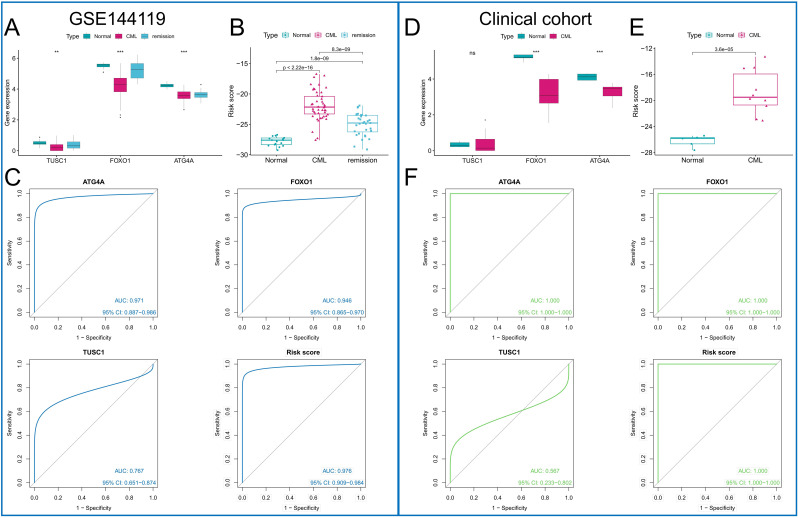
Validation of the diagnostic value of the diagnostic ARGs. **(A, D)** Differences in the expression of the three diagnostic ARGs between CML samples and normal samples in the GSE144119 cohort **(A)** and our clinical cohort **(D)**. **(B, E)** Differences in the risk score between CML samples and normal samples in the GSE144119 cohort **(B)** and our clinical cohort **(E)**. **(C, F)** ROC curve analysis was used to evaluate the diagnostic value of the three ARGs and the risk score in the GSE144119 cohort **(C)** and our clinical cohort **(F)**. (ns: P > 0.05; **P < 0.01; ***P < 0.001).

The diagnostic value of the three ARGs was validated in two additional cohorts. Downregulation of all three diagnostic ARGs was observed in CML samples in the GSE144119 cohort (**Figure 7A**), as well as in our clinically independent cohort (**Figure 7D**), in which the difference in TUSC1 expression between CML and normal samples did not show statistical significance, possibly due to the small sample size. In addition, CML samples had higher risk scores than normal samples in both cohorts (**Figures 7B, E**). The results of the ROC curve analysis showed that the AUC values of ATG4A, FOXO1, TUSC1, and the risk score model were 0.971, 0.946, 0.767, and 0.976, respectively, in the GSE144119 cohort (**Figure 7C**) and 1, 1, 0.567 and 1, respectively, in our clinical cohort (**Figure 7F**). The specificity of the risk score model in the GSE144119 cohort was 0.971 and the sensitivity was 0.914. In our clinical cohort, the specificity and sensitivity of the risk score model were both 1. These results validate the high diagnostic value of the three ARGs in CML. In the GSE144119 cohort, which contained data from samples in remission after TKI treatment, the expression of the three diagnostic ARGs in the remission samples returned to an expression level close to that of normal samples (**Figure 7A**), and the risk scores were also lower than those of the newly diagnosed CML patients (**Figure 7B**), indicating that these diagnostic ARGs can be used to evaluate the treatment effect of CML.

### Analysis of the differential diagnostic value of ARGs

**Figure 8:**
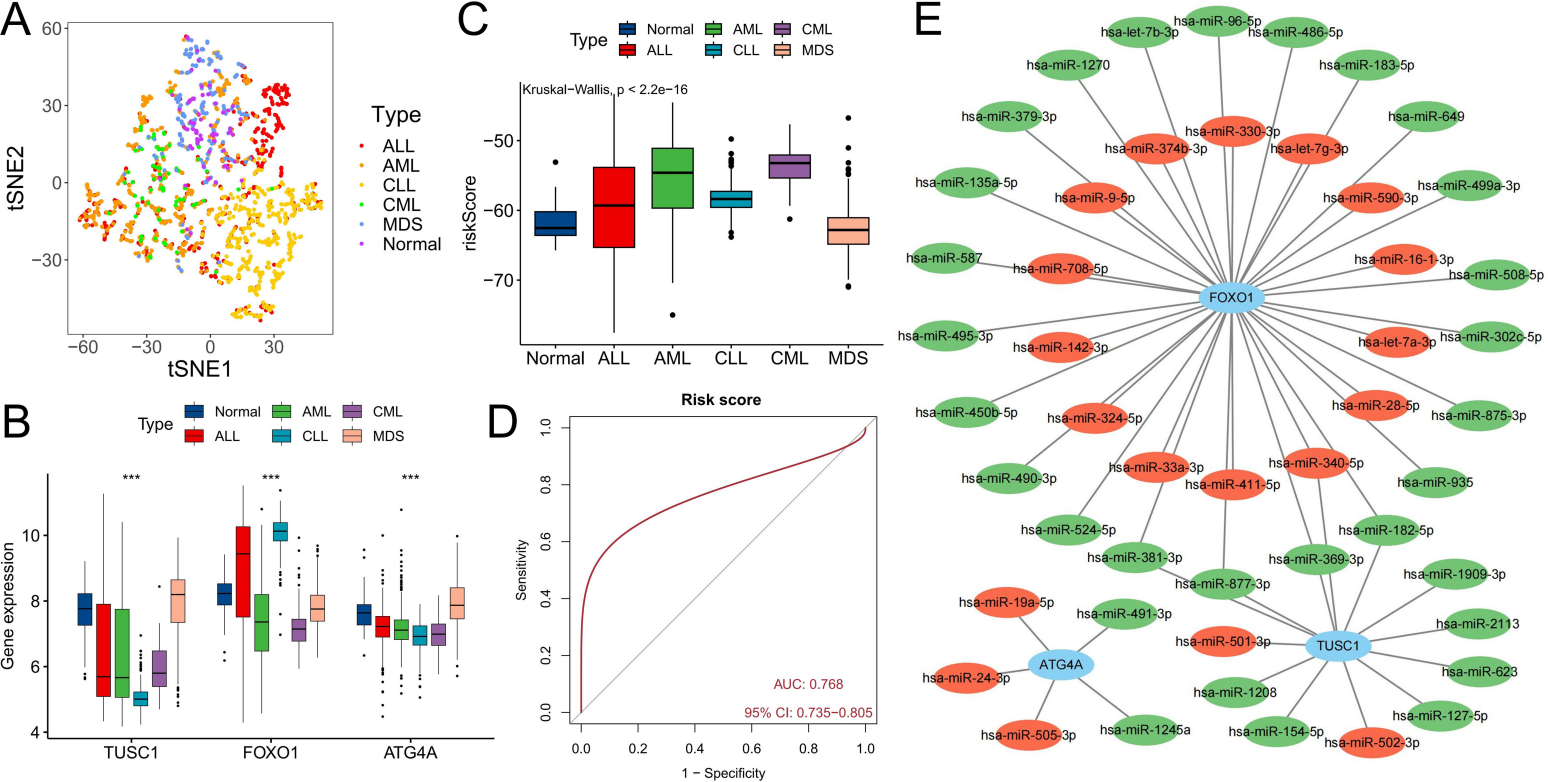
Differential diagnostic value of the three ARGs in CML and other hematological malignancies. **(A)** The t-SNE plot shows the clustering characteristics of CML, AML, CLL, ALL, MDS and normal samples based on the expression of three diagnostic ARGs (FOXO1, TUSC1, ATG4A). The horizontal axis (t-SNE 1) and the vertical axis (t-SNE 2) are the principal components after dimensionality reduction, with no unit dimension, used to visualize the local structure of high-dimensional data. Different colored dots represent the corresponding samples. **(B)** Differences in the expression of three diagnostic ARGs among CML, AML, CLL, ALL, MDS, and normal samples. **(C)** Differences in risk scores among CML, AML, CLL, ALL, MDS, and normal samples. **(D)** ROC curve analysis of risk scores in patients with CML and other hematological malignancies. **(E)** Regulatory network of miRNAs and the three diagnostic ARGs; red indicates that miRNA expression is upregulated in CML samples, and green indicates that miRNA expression is downregulated. (***P < 0.001).

The GSE13159 cohort also included 750 ALL samples, 542 AML samples, 448 CLL samples, and 206 MDS samples. We analyzed the expression characteristics of the three diagnostic ARGs in these samples. The t-SNE algorithm was used to cluster all samples based on the expression of three diagnostic ARGs. In addition to overlapping with the distribution of some AML patients, CML patients exhibited distinct differences from patients with other hematologic malignancies (P<0.05) (**Figure 8A**). Compared with those in the other five types of samples, the expression levels of the three ARGs in the CML samples were low (P<0.05) (**Figure 8B**), while the risk score was the highest (**Figure 8C**). ROC curve analysis revealed that the risk score could accurately distinguish CML from other hematological malignancies (area under the curve (AUC)=0.768) (**Figure 8D**). Finally, we constructed a miRNA regulatory network in which miRNAs upregulated in CML cells may suppress ARG expression through their binding to ARGs. Red indicates upregulated miRNA expression in CML samples, while green signifies downregulated miRNA expression (**Figure 8E**). The establishment of this network also offers potential insights into the regulatory mechanism of ARGs.

## Discussion

Autophagy is a catabolic process that plays a dual role in tumor suppression and promotion (9), and its activation may help tumor cells adapt to cellular stress and, in some cases, may also lead to cell death. Induction of autophagy is considered to be an effective way to prevent cancer (5). Through selective autophagy, cells can remove damaged mitochondria that produce reactive oxygen species, thereby preventing the occurrence of DNA mutations (27). In addition, autophagy may also contribute to the survival of tumor cells. Studies have shown that the combination of chemotherapy drugs and autophagy inhibitors can kill tumor cells and inhibit tumor occurrence more than chemotherapy drugs alone (28). At present, the use of autophagy as a target for intervention has been controversial. Similar to that in other tumors, autophagy plays two roles in CML.

In this study, we analyzed the autophagy level in CML samples and the value of ARGs in CML diagnosis and treatment evaluation through transcriptome data. The results of multiple sets of data analysis showed that the autophagy score of CML samples was significantly reduced, and the autophagy score further decreased with the progression of CML. In addition, nonresponders had lower autophagy scores than did those who responded to TKI treatment. Therefore, CML cells may promote malignant proliferation and chemotherapeutic resistance by inhibiting autophagy. To better understand the regulatory mechanism of autophagy in CML, we analyzed the relationship between the autophagy score and signaling pathways and the immune microenvironment and found that a lower autophagy score was associated with greater proliferation pathway activity and lower apoptosis pathway activity, which was consistent with the correlation trend we observed previously. Similarly, CML patients with different autophagy scores had different immune characteristics. A greater proportion of patients with high autophagy scores exhibited Treg infiltration, while patients with low autophagy scores exhibited increased γδT cell and plasma cell infiltration and increased PD-1 expression. By further WGCNA, we found that the coexpressed genes that were significantly positively correlated with the autophagy score were mainly enriched in the cytokine–cytokine receptor interaction signaling pathway. Based on these biological characteristics, it is reasonable to speculate that the heightened autophagy activity of CML cells may induce apoptosis. Consequently, CML samples exhibit lower autophagy scores, which are indicative of a poor prognosis. In an attempt to address this situation, the infiltration of killer cells such as γδT cells is increased; however, their functional exhaustion occurs due to the upregulation of immune checkpoints such as PD-1 (29). However, in CML patients with high autophagy scores, CML cells may regulate the infiltration and activity of Tregs through the release of autophagy-induced cytokines, resulting in immunosuppression (30, 31). Previous studies have shown that the proportion of Tregs in newly diagnosed CML patients is significantly increased, indicating that Tregs may be involved in the occurrence and development of CML (32). Another study confirmed that Tregs expressing Tnfrsf4 promoted immune escape from CML stem cells (33). Therefore, the immune characteristics of patients with different autophagy scores show significant heterogeneity and may play a corresponding role in tumor growth and immune escape at various stages.

The expression characteristics and biological functions of the ARGs were further analyzed. More DEARGs were downregulated in the CML samples. In addition to regulating autophagy, DEARGs also participate in a variety of signaling pathways closely related to the occurrence and development of cancer. Moreover, the expression of ARGs with the same expression trend showed a significant positive correlation, suggesting that there may be coordination in their functions. The identification of molecular subtypes is also conducive to a deeper understanding of the individual characteristics of CML patients. The significantly increased infiltration of CD8+ T cells and the upregulated expression of multiple immune checkpoints in the C1 subtype reconfirmed the existence of significant immunosuppression in CML patients, and such patients may benefit from immunotherapy. In addition, the C1 subtype is also more sensitive to multiple TKIs. These findings are helpful for advancing our understanding of CML disease states and pathological mechanisms and for guiding personalized clinical treatment. Machine learning models are beneficial for improving the diagnosis, risk assessment, and treatment management of CML patients (34). In this study, we identified three CML diagnostic ARGs, FOXO1, TUSC1, and ATG4A, by three machine learning algorithms, all of which were downregulated in CML samples. Previous studies have shown that the tyrosine kinase BCR-ABL in CML cells activates multiple signal transduction pathways, including the PI3K/AKT signaling pathway, thereby inactivating FOXO transcription factors. TKI-induced G1 arrest in CML cells is mediated by inhibition of the PI3K/AKT pathway and FOXO reactivation (35). Therefore, the downregulation of FOXO1 expression is associated with TKI insensitivity and BCR-ABL enhancement. Tumor suppressor candidate 1 (TUSC1) is a tumor suppressor gene that reduces tumor cell growth *in vitro* and tumor growth *in vivo* (36). ATG4A is a classical autophagy-related gene (37, 38), and a reduction in its expression indicates a reduction in autophagic activity. The downregulation of the expression of these two genes favored tumor cell growth. The diagnostic value of the three diagnostic ARGs was confirmed in the analysis cohort, validation cohort and our additional clinical reality cohort, and the diagnostic value of the risk score model constructed by combining the three ARGs was further improved. In addition, the expression level of ARGs can also indicate the response to treatment in CML patients. More importantly, these three ARGs can also be used for the differential diagnosis of CML from other hematological malignancies. Previous studies on CML biomarkers primarily focused on BCR-ABL transcript levels or somatic mutations (1), which lack specificity in distinguishing CML from other hematologic malignancies (e.g., AML with monocytic differentiation or MDS with fibrosis). In contrast, our machine learning-based ARGs (FOXO1, TUSC1, ATG4A) exhibited remarkable specificity. For instance: FOXO1 is downregulated in CML but shows no significant change in AML or CLL, unlike previous pan-cancer autophagy markers (e.g., BECN1) that lack disease specificity (7). The risk score model demonstrated an AUC of 0.768 for differentiating CML from AML/CLL/ALL/MDS, indicating a relatively good diagnostic value. These results fully showed the value of ARGs in the diagnosis and treatment evaluation of CML.

In summary, through this study, we revealed the characteristics of autophagy in CML from the perspective of transcriptomics, and these results are conducive to a better understanding of the biological role of autophagy in CML. The significant differences in the immune microenvironment among patients with different autophagy scores also suggest that immunosuppression is an important factor in the disease progression of CML. In addition, the identification of autophagy-related molecular subtypes is conducive to the establishment of personalized treatment regimens for CML patients. The ARGs identified by the use of a variety of machine learning algorithms and the validation of multiple cohorts have reliable diagnostic value. However, our study has several limitations, including the small sample size for clinical validation and the lack of experimental validation. In addition, larger sample sizes and multicenter data are required to assess the variations in ARG expression across different stages of CML, as well as to determine the optimal cutoff value for machine learning models to enhance their translation and application in clinical diagnosis. Furthermore, emphasis should be placed on genetic testing and quantification methods for diverse datasets while optimizing testing approaches to improve detection efficiency. In the future, we will expand our sample collection and explore more in-depth autophagy regulatory mechanisms through *in vivo* and *in vitro* experiments.

## Conclusion

In conclusion, our study demonstrated a decrease in autophagy in CML samples at the transcriptome level and revealed that autophagy may promote immunosuppression by regulating cytokines and Tregs. Autophagy-related molecular subtypes are helpful for guiding the clinical treatment of CML. The identification of ARGs by a variety of machine learning algorithms has potential clinical application value.

## Data Availability

The data presented in the study are deposited in the GEO repository, accession number GSE13159, GSE144119.
